# Are Depressive Symptoms in Obstructive Sleep Apnea Attributable to a Syndrome of Dysregulation of Rhythms and Hyperactivity (DYMERS)?

**DOI:** 10.3390/jcm13154396

**Published:** 2024-07-27

**Authors:** Diego Primavera, Elisa Cantone, Gregorio Marco Cannizzaro, Chiara Sanna, Stefania Redolfi

**Affiliations:** 1 Department of Medical Sciences and Public Health, University of Cagliari, Monserrato Blocco I (CA), 09042 Cagliari, Italy; diego.primavera@tiscali.it (D.P.); stefania.redolfi@unica.it (S.R.); 2 Research Center of Sleep Disorders, University Hospital D. Casula Monserrato, Monserrato, 09042 Cagliari, Italy; g.cannizzaro@studenti.unica.it (G.M.C.); c.sanna2@studenti.uniss.it (C.S.)

**Keywords:** obstructive sleep apnoea, mood disorder, bipolar disorder, quality of life, rhythm dysregulation, sleep disorder

## Abstract

**Background:** Obstructive sleep apnea (OSA) is characterized by repeated airway obstructions during sleep, causing hypopnea, apnea, intermittent hypoxia, and sleep fragmentation. The severity of OSA is measured using the apnea–hypopnea index (AHI), with AHI ≥ 5 indicating OSA. This study aims to assess the frequency and type of depressive disorder characteristics of OSA patients and to evaluate the impact on quality of life, also considering the presence of hyperactivity. **Methods**: A case-control study using OSA patients referred to Cagliari’s sleep disorder center. Controls were matched by age and sex from community databases. OSA diagnoses were made with AHI > 15. Depressive episodes were identified using BDI-SF, and H-QoL (Health related Quality of Life) was measured with the SF-12, focusing on item 10 for hyper-energy. **Results**: The clinical sample (*n* = 25) had a higher frequency of depressive episodes (36%) compared to controls (7% and 4%). Depressed OSA patients had worse H-QoL and higher hyper-energy scores, but the additional burden from depression was relatively low. **Conclusions**: The OSA sample has a higher frequency of depressive episodes compared to the general population. Depressive episodes in OSA patients are linked to higher scores on item 10 of the SF-12, indicating hyper-energy despite lower overall quality of life scores. While OSA significantly impacts quality of life, the additional burden from depression is less severe than in other chronic diseases. These findings suggest that depressive episodes in OSA may be related to rhythm dysregulation and hyperactivity (DYMERS).

## 1. Introduction

Obstructive sleep apnea (OSA) is characterized by repetitive upper airway obstruction during sleep, resulting in cycles of partial (hypopnea) or complete (apnea) cessation of airflow associated with intermittent hypoxia, sleep fragmentation, and intra-thoracic pressure changes [1]. The degree of OSA severity has traditionally been measured by the apnea–hypopnea index (AHI), which is the average number of apneas and hypopneas per hour of sleep. OSA is the most common sleep-related breathing disorder affecting nearly one billion people, with approximately half having moderate to severe disease (AHI ≥ 15) [2], which has significant implications for cardiovascular health, driving safety, mental disorders, and quality of life [3].

The association between OSA and mental health disorders has recently gained significant attention. Depression is highly prevalent in OSA patients, and its presence can exacerbate sleep disturbances and decrease treatment adherence. A recent meta-analysis and systematic review estimated that the prevalence of depressive episodes in OSA patients was as high as 35% [4]. Moreover, Kaufman et al. showed that OSA patients were 3.11 times more likely to be depressed, 2.75 times more likely to have suicidal thoughts, and 2.88 times more likely to experience severe mental stress compared to individuals without OSA [5].

A recent large database study using Mendelian randomization found that genetic predisposition for major depressive disorder (MDD) increases the risk of OSA and that genetic predisposition for OSA may have a causal effect on ADHD. This study found no association with other mental health conditions [6]. Other recent studies using similar methodologies have confirmed the association of OSA with MDD [7,8]. Other studies found OSA associated with bipolar disorder (BD) [9], with great variability in different studies of the co-morbidity frequency of OSA in BDs, i.e., from 2.9% to 69% [10]. A meta-analysis reported an average 24.5% prevalence of OSA in people with BP in clinical settings [11]. This discrepancy could be due to the methods of identification of depressive episodes and the consequent diagnoses of either MDD or BD. There is a wide debate about the clear distinction between these disorders, with opposing views of the American Psychiatric Association, which marked a strict distinction in the DSM-5 classification [12] and the so-called neo-Kraepelinian view, which encompasses the entire spectrum of bipolar disorders, including MDD and BD [13,14,15]. This view also considers the spectrum as a continuum between clear clinical pictures, subthreshold forms, and hyperergic hyperthymic temperaments that are not necessarily pathological [16,17,18]. This debate is particularly interesting in the field of OSA because a syndrome characterized by hyperactivity, stress, and loss of biological rhythms (DYMERS) has recently been described as an area of shared risk between different pathologies, especially bipolar spectrum disorders. DYMERS is a proposed condition characterized by the dysregulation of behavioral rhythms, affecting sleep patterns, eating habits, and social interactions. It is considered an intermediate state between normal adaptive energy increase and hyperactivity during manic episodes. DYMERS is associated with a continuous stimulation of stress hormones and may lead to depressive episodes, burnout, and altered immune responses. It is seen as a vulnerable condition that can evolve into other disorders, such as bipolar disorder, depending on individual susceptibility and stress levels. [19,20,21]. In OSA, parasympathetic activation is observed during apneas, which causes phases of hyperactivation with consequent cardiovascular risks [22]. Furthermore, transcutaneous oxygen saturation measured overnight was found to be negatively associated with hyperactivity/impulsivity scores in the SNAP-IV questionnaire of the ADHD symptom scale in children with sleep-disordered breathing [23]. The dysregulation of biological rhythms and the consequent dysregulation of behavioral rhythms (sleeping, eating, having social contacts) is a characteristic of OSA [24] to the point that this condition has been defined as a disorder of biological rhythms [25].

It has recently been seen that item 10 of the SF-12 scale [26,27], one of the most used tools in the world to assess health-related quality of life, can measure hyperactivity [28] with good reliability with an instrument such as the Mood Disorder Questionnaire [29] that was initially conceived as a screening tool for BD but later turned out to be a good tool for identifying DYMERS syndrome [30,31].

Starting from these assumptions, our work aims to measure the frequency of depressive episodes in a sample of patients suffering from OSA and referring to a clinical center and compare the frequency with that of a control sample, without the condition, taken from the database of a community study. With a similar methodology, the study aims to measure the difference in the perception of quality of life in patients with OSA and a balanced sample of the general population to compare the burden of OSA in worsening the quality of life with that of other chronic pathologies and to measure the impact of depression on quality of life. Finally, we will try to verify, at least in a preliminary way, whether the hyperactivity measured with item 10 of SF-12 characterizes depressive episodes in OSA. Our hypothesis is that, at least in part, the depressive episodes in OSA are attributed to a syndrome of dysregulation of rhythms and hyperactivity attributable to the historical spectrum of bipolar disorders.

## 2. Methods

This is a case-control study. The case sample consists of consecutive patients referred for treatment at the sleep disorder center of the University Hospital of Cagliari over the age of 18, without limitations relating to sex. Patients with non-affective psychotic pathologies were excluded. Control groups: two control groups were drawn from two community databases, the first allowing the prevalence of depressive episodes by screening methods similar to that adopted in the clinical sample [32], the second allowing the measure of the prevalence of major depressive episodes by means of a structured clinical interview according to DSM-IV criteria [33] and the measure of the health-related quality of life by means of SF-12 [34]. For each clinical case, a matched block with people of the same age (or in the case of the first control sample ±1 year) and a sex-healthy control was created. From each cell, four controls were randomly selected. Once drawn out, the subject was automatically excluded from the remaining blocks.

### 2.1. Measures

#### 2.1.1. Clinical Diagnosis of OSA

Subjects eligible for the study were selected from patients referred to the sleep disorders center of the University Hospital of Cagliari for suspected OSA. After the initial clinical evaluation, all patients underwent a home sleep apnea test, polygraphy type III (Embla System, Natus Medical Inc., Oakville, Ontario L6H 5S1 Canada). The following parameters were recorded during the sleep study: nasal pressure and oral thermistor for oronasal flow assessment, thoracic and abdominal respiratory inductance plethysmography belts for evaluation of respiratory efforts and for detection of thoraco-abdominal asynchronism or paradox, pulse-oximetry, 1-lead EKG, and body position. A trained physician scored polysomnographic recordings manually using Remologic software (version Embla 3.4.1). Respiratory events were scored using the latest AASM criteria [35] as follows: apnea was defined as a drop of at least 90% of airflow from baseline, lasting 10 s or longer, and hypopnea as a ≥30% drop of airflow lasting at least 10 s, associated with either arousal or a ≥3% oxygen saturation drop. The average number of apneas and hypopneas per hour of sleep (AHI) was calculated. In accordance with the American Academy of Sleep Medicine [36], the diagnosis and classification of OSA severity was performed based on the AHI. We prospectively included patients with AHI > 15.

In the general population sample, the presence of chronic diseases was revealed by an anamnestic form. Given the different methods of ascertainment between cases and controls and the delay in the diagnosis of OSA, it is possible that there will be some unrecognized cases among the controls. The implications of this possibility, which would, in any case, be entirely to the advantage of the null hypotheses, will be discussed within the limits.

#### 2.1.2. Diagnosis of Depressive Episodes

The assessment of depressive symptoms in the first control sample (“A”) was carried out using the BDI-SF, a short form of the BDI composed of 13 items [37]. The BDI-SF appears to have a level of internal consistency (coefficient alphas) comparable to that of the long form [38]. Pearson product–moment correlation coefficients between the BDI and the BDI-SF have ranged from 0.89 to 0.97, indicating that the short form is an acceptable substitute for the long [39].

#### 2.1.3. Measurement of Health-Related Quality of Life

The total score of the self-administered Italian version [40] of the Short Form Health Survey, 12-item version (SF-12) [26,27], was used for measuring the perception of the health-related quality of life (H-QoL). Item 10 of the SF-12 was used as a measure of the hyperactivity in depressed and not-depressed people in cases and the control group.

## 3. Statistical Analysis

Data were collected anonymously using subject ID numbers. The difference between the frequencies in depressive episodes in the cases and controls was measured using chi-square, with Yates correction if required. The attributable burden of OSA on the worsening of H-QoL was calculated as the difference between the overall score of SF-12 in a control group matched for sex and age within the community and that of the study sample. Differences in H-QoL score among subgroups were carried out by means of one-way analysis of variance (ANOVA). Furthermore, the burden of OSA in worsening H-QoL was compared to the burden of other diseases, as calculated in previous case-control studies from the same dataset used to select the control groups [41,42,43,44,45,46,47,48,49]. Subsequently, the specific and adjunctive burden related to depressive episodes was calculated as the difference in the SF-12 scores of individuals with OSA without depression and those with OSA and depression. A similar method was used in prior studies that utilized the same dataset to draw control groups. Thus, we compared the burden attributed to current co-morbid major depressive episodes in several chronic diseases to those observed in OSA.

## 4. Results

Table 1 shows the characteristics of the clinical sample (N = 25) and the two control groups (N = 100) perfectly balanced for sex and age because of the randomization by blocks. The clinical sample had a preponderance of male subjects (64%) and a medium-high average age of 63.12 years. The case group was obese with a body mass index of 31 ± 7 kg/m^2^ and had classic OSA co-morbidities: 76% had artery hypertension, 64% had dyslipidemia, 36% had diabetes, 32% had chronic ischemic heart disease, and 16% had atrial fibrillation. OSA was severe (AHI 49 ± 25) and associated with severe nocturnal oxygen desaturation (time with SpO_2_ < 90% of 33 ± 23% of the total recording time). The frequency of depressive episodes in the clinical sample was higher than in both the control group that used a similar screening tool (36% vs. 7%, OR = 7.47, CI 95% 2.4–22.9) as well as in the control group adopting a clinical diagnosis according to DSM-IV (36% vs. 4%, OR = 18.20, CI 95% 4.4–74.5). Male OSA patients were at risk of depression compared to male subjects of both control groups, 31.25% versus 3.12% if we consider the control sample that used a screener to identify the depressive episodes (OR = 14.09, CI 95% 2.4–81.9) and 3.12% if we consider the control sample that used a clinical diagnosis (OR = 14.09, CI 95% 2.4–81.9). Females OSA patients, on the other hand, although recording a higher frequency than the two control groups, reached a statistically significant difference only compared to the control sample that used the clinical investigation (44.4% vs. 5.5%, OR = 28.00 CI95% 4.4–74.5). Table 2 shows that OSA patients with depression have a worse perception of quality of life than those without depression, but the difference is not statistically significant (29.44 ± 2.54 vs. 31.12 ± 3.79, *p* = 0.239). In controls, the presence of a depressive episode produces a worsening of the level of perception of quality of life (38.50 ± 5.2 versus 31.5 ± 4.15, *p* = 0.003) (however, the small sample size is at the limits of the applicability of the Kruskal–Wallis test). With regards to item 10 of the SF12, this was higher in OSA patients with depression compared to those without depression (5.00 ± 0.66 vs. 3.43 ± 1.95, one-way ANOVA, 1, 24 df, F =8.837, *p* = 0.007); an opposite result, i.e., a lower SF-10 score in those with depression was found in the community sample (2.75 ± 0.43 vs. 4.31 ± 1.10, H = 7.6812, *p* = 0.006 Kruskal–Wallis test).

As shown in Table 3, the attributable burden on worsening QoL of OSA is like that found in major depressive disorder, multiple sclerosis, carotid atherosclerosis, and systemic lupus erythematosus. The attributable burden on worsening QoL of OSA was higher with respect to chronic diseases with a more unfortunate outcome probability, such as solid cancers (attributable burden 4.67 ± 6.64 vs. 7.70 ± 4.84, *p* = 0.030), or other chronic diseases such as Wilson’s disease (attributable burden 4.4 ± 1.7 vs. 7.70 ± 4.84, *p* < 0.0001 ), Celiac disease (attributable burden 2.4 ± 1.0 vs. 7.7 ± 4.84, *p* < 0.0001), or chronic psychiatric disorders such as obsessive–compulsive disorder (attributable burden 2.9 ± 6.0 vs. 7.70 ± 4.84, *p* < 0.0001), post-traumatic stress disorder (attributable burden 3.9 ± 1.0 vs. 7.70 ± 4.84, *p* < 0.0001), or specific phobia (attributable burden 0.4 ± 4.9 vs. 7.70 ± 4.84, *p* < 0.0001).

The component attributable to co-morbidity with depression in the worsening of the quality of life is extremely low in OSA; in fact, it is the lowest among the pathologies considered, and the difference due to the presence of depression in lowering the perception of quality of life is statistically significant against systemic lupus erythematosus (decrease in the SF-12 score attributable to co-morbidity with depression 9.43 ± 5.10 in SLE versus 1.68 ± 4.0 in OAS, *p* < 0.0001), fibromyalgia (decrease in the SF-12 score attributable to co-morbidity with depression 4.77 ± 5.76 versus 1.68 ± 4.0 in the OAS, *p* = 0.014), and solid cancers (decrease in the SF-12 score attributable to co-morbidity with depression 10.1 ± 5.7 versus 1.68 ± 4.0 in the OAS, *p* <0.0001), as shown in Table 4.

## 5. Discussion

Our study confirms that patients suffering from OSA have a much higher frequency of depressive episodes identified by a screening test than the general population. These depressive episodes are characterized by a male predominance by a paradoxical increase in hyperactivity assessed by item 10 of the SF-12, while the perception of quality of life according to the general score on the SF-12 questionnaire is the same in OSA patients with and without depression. The score on item 10 alone in the control sample with depression is lower than in controls without depression. Although OSA appears to have a strong impact on compromising the quality of life, and this occurs to a greater extent than diseases with more unfortunate outcomes such as solid cancers or chronic psychiatric pathologies such as obsessive–compulsive disorder, the co-morbidity with depression worsens the impact on quality of life but to a lesser extent than in many other diseases. In conclusion, we can say that what is identified as depression as per the screener tools presents paradoxical characteristics of hyper-energy, has a greater frequency in males than in women in relation to what is expected in major depressive disorder, and impacts the quality of life in the negative sense but to a much lesser extent than in other chronic diseases. All of this is in a general framework of dysregulation of behavioral and biological rhythms typical of OSA.

Robust research has underlined how screening for mental health conditions conducted with paper and pencil tests identifies people with psychosocial distress but does not always coincide with those to whom the specific clinical diagnosis is attributed. This has been reported both in the case of major depressive disorder [50] and in bipolar disorders [51,52]. Another possible error due to the use of pen and paper tests is that these identify the current frequency of depressive episodes, but in diagnostic terms, a depressive episode can be part of both a lifetime diagnosis of bipolar disorder and major depression [53].

In our study, this possible bias was balanced by the fact that we compared our experimental sample both with a control sample in which a screener had been used and with a control sample that had used a clinical interview to identify depressive episodes. We can, therefore, state that the emerging differences are typical of depression in OSA and not the result of bias due to the use of a screening test. It must be underlined from this point of view that the presence of a hyperactivity/hyper-energy component suggests that the episodes identified in OSA may belong to the so-called bipolar spectrum and that the higher prevalence in males can be interpreted in the same sense [54]. However, the impact of these episodes on the worsening of quality of life is present, but it is less than expected if they were part of a full diagnosis of bipolar disorder II. It has, in fact, been highlighted that bipolar type II is associated with a stronger, poorer quality of life not only against healing controls but even compared to type I bipolar disorder and during periods of euthymia without residual symptoms [55].

Based on previous studies by our group [21,56], the following three different levels of hyperactivation/hyper-energy can be defined, ranging from an adaptive response to psychological distress to conditions of dysregulation of biorhythms and, therefore, up to frank mental disorders:

(1) The first level is an adaptive increase in energy in response to exceptional demands. Typical of a person who prepares to face a difficult task but who pushes himself towards success. For example, in trained athletes, the increase in adaptive energy can be strong; in fact, some sports champions report having achieved excellent performances even if they had not slept the day before the performance precisely because they were in a state of hyperactivation [57];
(2) The second is an increase in energy linked to distress and is associated with a dysregulation of biological and social rhythms (such as sleep, social relationships, and eating rhythms) [58]. This condition is typical of strong and persistent stress over time or vice versa of conditions in which stress is combined with dysregulation of rhythms, as in the stress of shift workers [59]. In these cases, the increase in energy can be focused on the goal/problem that produces the stress but is ineffective, which is typical of professional burnout. In fact, it is known that hyperactivation accompanies stress syndromes [60,61]. Many of those positive on the screener for major depression in OSA could belong to this category, which is, however, associated with distress, compromised quality of life and can represent a serious risk condition for further mental disorders [49];
(3) The third level is hyperactivation and increased energy in episodes of hypomania or frank mania. In this context, hyperactivation ends up being out of the individual’s control and ends up no longer being aimed at adaptive objectives (hyperactivation in burnout stress, however, as mentioned in the previous point, although ineffective, is still focused on overcoming difficulties). For an episode of mania to occur, the environmental risk factors must be associated with specific personal risk conditions, perhaps of a genetic nature. A predisposition to hyperactivity could be the substrate of the disorder if triggered by severe stress.

Our results seem to suggest that at least some of the mood impairment conditions highlighted in OSA by positivity to the depression screener may, in reality, be rhythm dysregulation and hyperactivity syndromes (DYMERS), which, although associated with a lower quality of life impairment than that of a full depressive episode (in the course of major depressive or bipolar disorder) still represent an area of strong risk for the evolution of more serious conditions.

Our results will, however, need to be confirmed by studies centered on more accurate syndromic assessments that study large numbers of patients with OAS over time.

## 6. Limits

Our study shows objective limitations: first of all, the small size of the clinical sample, then the fact that the control groups were taken from databases of studies in the general population in which the measurement of the absence of OSA was much less accurate than in the sample of cases. In fact, it is possible that among the controls, there was the presence of some unrecognized cases, given the delay in diagnosis of OSA. However, this is an infrequent condition (5–10% of the general population) and an elderly sample (over 60 years on average) where the risk of non-recognition decreases with age. The implications of the possibility of contamination with false positives in the controls, however, would be entirely to the advantage of the null hypotheses. Therefore, the effect would have been to annul the hypotheses that have been proven. However, future studies will have to take these aspects into account.

## 7. Conclusions

Patients with obstructive sleep apnea (OSA) have a significantly higher frequency of depressive episodes characterized by increased hyperactivity, which significantly impacts their quality of life. The presence of hyperactivity suggests that these depressive episodes may belong to the bipolar spectrum, particularly given the higher prevalence in males. OSA is a condition closely related to sleep and eating disorders, and the study suggests that at least some of the mood impairment conditions highlighted in OSA by positivity to the depression screener may be DYMERS, which, although associated with a lower quality of life impairment than that of a full depressive episode (in the course of major depressive or bipolar disorder) still represent an area of great risk for the evolution of more serious conditions. These suggestions need to be confirmed by studies centered on more accurate syndromic assessments and large numbers of patients with OAS followed over time.

## Figures and Tables

**Table 1:** Study samples and frequencies of depressive episodes in cases and two control groups.

	OSA N = 25	Control Group A * Randomized by Blocks (4/1) From a Community Data Bank N = 100			Control Group B ** Randomized by Blocks (4/1) From a Community Data Bank N = 100		
Age	63.12 ± 11.96	63.46 ± 12.28	Matching		63.12 ± 11.96		Matching
Sex M	16 (64%)	64 (64%)	Matching		16 (64%)	64 (64%)	Matching
Screened Positive for Depression	9/25 (36%)	7/100 (7%)	Chi-square 15.070 *p* < 0.0001	OR 7.47 CI 95% (2.4–22.9)	4/100 (4%)	Chi-square Yates corrected Chi-square 18.078 *p* < 0.0001	OR = 18.20 CI 95% (4.4–74.5)
Positives for Depressive Episodes Females	4/9 (44.4%)	5/36 (13.8%)	Chi-square Yates corrected 2.509 *p* = 0.113	OR 4.96 CI 95% (0.98–25.0)	2/36 (5.5%)	Chi-square Yates corrected 8.798 *p* = 0.002	OR = 28.00 CI 95% (4.4–74.5)
Positives for Depressive Episodes Males	5/16 (31.25%)	2/64 (3.12%)	Chi-square Yates corrected 9.403 *p* = 0.002	OR 14.09 CI 95% (2.4–81.9)	2/64 (3.12%)	Chi-square Yates corrected 9.423 *p* = 0.012	OR 13.6 CI 95% (2.4–81.9)

* Group A: prevalence of depressive episodes by screening methods similar to that adopted in the clinical sample, ** prevalence of major depressive episode by means of a structured clinical interview according to DSM-IV criteria.

**Table 2:** Comparison of SF-12 total score and item 10 SF-12 score in people with and without depressive episodes in cases and in controls (sample “B”).

	OSA with Depression N = 9	OSA Without Depression N = 36		*p*	Major Depressive Episode Control B N = 4	Without Major Depressive Episode Control B N = 96	Kruskal–Wallis	*p*
SF-12 Total	29.44 ± 2.54	31.12 ± 3.79	ANOVA one-way 1,24 df F = 1.463	0.239	31.5 ± 4.15	38.50 ± 5.2	*H* = 8.3885	0.003
SF-12 Item 10	5.00 ± 0.66	3.43 ± 1.95	ANOVA one-way 1,24 df F = 8.837	0.007	2.75 ± 0.43	4.31 ± 1.10	*H* = 7.6812	0.006

**Table 3:** Attributable burden on worsening QoL in patients with OAS in comparison to the attributable burden due to other chronic diseases.

Disease	SF-12 (Mean ± SD)	Attributable Burden on QoL	Comparison with OAS (One-Way ANOVA)
Major Depressive Disorder [34]	33.8 ± 9.2	5.6 ± 3.6 (N = 37)	F = 3.779, df 1, 60; *p* = 0.057
Multiple Sclerosis [42]	29.5 ± 7.3	7.0 ± 3.5 (N = 201)	F = 0.810, df 1, 225; *p* = 0.369
Wilson’ Disease [41]	33.8 ± 9.0	4.4 ± 1.7 (N = 23)	F = 34.949, df 1, 47 *p* < 0.001
Carotid atherosclerosis [44]	30.6 ± 8.1	6.2 ± 5.0 (N = 46)	F = 1.490, df 1, 70; *p* = 0.226
Celiac Disease [43]	35.83 ± 5.72	2.4 ± 1.0 (N = 60)	F = 66.231, df 1, 84; *p* < 0.001
Obsessive–Compulsive Disorder [45]	35.4 ± 6.9	2.9 ± 6.0 (N = 88)	F = 13.278, df 1, 112; *p* < 0.0001
PTSD [47]	36.3 ± 6.1	3.9 ± 1.0 (N = 26)	F = 13.038, df 1, 46, 56 *p* < 0.0001
Specific Phobia [46]	38.3 ± 5.2	0.4 ± 4.9 (N = 28)	F = 29.654 df 1, 52, 58 *p* < 0.0001
Solid cancer [49]	32.34 ± 6.764	4.67 ± 6.64 (N = 151)	F = 4.775; df 1, 175; *p* = 0.030
Systemic Lupus Erythematosus [49]	32.96 ± 7.09	5.37 ± 4.46 (N = 32)	F = 3.555; df 1, 56 *p* = 0.067
OAS	30.52 ± 3.34	7.70 ± 4.84 (N = 25)	PIVOT

**Table 4:** Attributable burden on worsening QoL in patients with OAS in comparison to the attributable burden due to other chronic diseases.

-	Attributable Burden to Major Depressive Disorder	One-Way ANOVA F (df)	*p*
Solid Cancers (N = 151) [48]	10.1 ± 5.7	50.806(1;175)	<0.0001
Multiple Sclerosis (N = 201) [42]	2.9 ± 7.4	0.698(1;225)	0.405
Fibromyalgia (N = 71)	4.77 ± 5.76	6.291(1;95)	0.014
Wilson’s Disease (N = 61) [41]	3.2 ± 7.9	0.878(1;85)	0.351
Celiac Disease (N = 60) [43]	3.4 ± 5.4	2.156(1;84)	0.146
Carotid Atherosclerosis (N = 46) [44])	3.4 ± 8.2	0.954(1;53)	0.333
Systemic Lupus Erythematosus (N = 31) [49]	9.43 ± 5.10	38.951 (1;55)	<0.0001
OAS (N = 25)	1.68 ± 4.0	Pivot	

## Data Availability

The datasets of this study will not be publicly available due to individual privacy rules.
